# The illusion confusion

**DOI:** 10.3389/fpsyg.2014.00231

**Published:** 2014-03-25

**Authors:** Clare Batty

**Affiliations:** Department of Philosophy, University of KentuckyLexington, KY, USA

**Keywords:** illusion, olfaction, olfactory misperception, olfactory illusion, object perception, olfactory objects, olfactory object perception

## Abstract

In Batty (2010b), I argue that there are no olfactory illusions. Central to the traditional notions of illusion and hallucination is a notion of object-failure—the failure of an experience to represent particular objects. Because there are no presented objects in the case of olfactory experience, I argue that the traditional ways of categorizing non-veridical experience do not apply to the olfactory case. In their place, I propose a novel notion of non-veridical experience for the olfactory case. In his (2011), Stevenson responds to my claim that there are no olfactory illusions. Although he agrees that it is natural—or at least commonplace—to think there are no olfactory illusions, he argues that there are and provides examples of them, many of which he suggests have analogs in the visual and auditory domains. In this paper, I examine the nature of the disagreement between us. I argue that Stevenson fails to argue against my conclusion that there are no olfactory illusions.

## Introduction

### Against olfactory illusions

Let me begin with an overview of my previous arguments^1^.

In Batty (2010a), I argue for a view according to which olfactory experience has representational content—that is, there is a way that the world appears to a subject when she has an olfactory experience. I set this discussion against suggestions previously in the literature (albeit brief) that olfactory experience may have no representational content—that is, that there is *no* way that the world appears to a subject when she has an olfactory experience^2^. These are views according to which olfactory experiences are “mere sensations,” or “raw feels.” I argue that driving these suggestions are differences between visual and olfactory phenomenology—that is, differences in what these two kinds of experiences are like for the subject. Visual experience is incredibly rich, seemingly offering up an array of three-dimensional objects. For this reason, the view that visual experience is world-directed—indeed directed at the objects in our environment—comes naturally to us, with the most common version of such a view the representational, or content, view. The case of olfactory experience is different. Although we might think that it presents a wealth of apparent properties, it does so with much less structure than its visual counterpart. As I have put it elsewhere, compared to visual experience, olfactory experience is “just plain smudgy.”

Despite this, I argue that there is a representational view of olfactory experience available and, as it turns out, we are able to draw that view from a certain debate about visual content. In the visual domain, there is significant disagreement about how visual experience represents that objects are thus and so. One view is that visual content is *abstract* and that your visual experience of a ripe tomato, for example, represents that there is “*something or other”* at a given location that it is red, round, and so on. This view is contrasted with the view that visual content is *object-involving*. On this view, the tomato itself (that *very thing*, there, before you) is a constituent of the content of your experience. That is, your experience represents that the *particular* tomato is at a given location and it is red, round, and so on. Unlike what the abstract view claims, your experience does not represent merely that “something or other” has those properties.

Drawing on several examples, I argue that olfactory experience does not represent particular objects in the way that some have argued vision does and, as a result, an object-involving view of olfactory experience is not available^3^. These examples all draw on what we might call day-to-day, or typical, olfactory experiences—namely, those that we have out in the world and not those that we might have in a controlled laboratory environment^4^. As most of us will never find ourselves in the laboratory environment, there remains an interesting question regarding the content of our typical olfactory experiences. Examining these typical cases olfactory experiences, I demonstrate that everyday olfactory experiences do not possess the robust spatial representation present in the visual case and, as a result, does not allow us to single out particular objects in our environment^5^. That is to say, unlike visual experience, olfactory experience does not reveal the particular objects that, in the case of veridical experience at least, bear the olfactory properties that it presents. This claim, I argue, is just the claim that olfactory experience does not achieve figure-ground segregation. Still, as I argue, an abstract view is a remarkably good fit for the olfactory case and suggestions that olfactory experience is merely sensational incorrectly cast an object-involving view as the only option for olfactory experience. The right view about the representational content of olfactory experience, I conclude, is one according to which it has a weak form of abstract content. In any circumstance, a given olfactory experience represents that there is something or other “here,” or “at” the perceiver, that has certain olfactory properties. I call this the *abstract view* of olfactory content.

In Batty (2010b), I turn to issues of misrepresentation with respect to the typical olfactory experience. In particular, I argue that the abstract view of olfactory content explains some of our intuitions about how olfactory experience can misrepresent the world. I point out that the notion of an olfactory hallucination is something that comes naturally to us while the notion of an olfactory illusion does not. This is reflected in the scientific literature on olfaction, in which reference to hallucination is common, but illusion rare. It has also been reflected in the philosophical domain—albeit in personal conversation and not in print—with a hesitancy in answering the question “*Are there olfactory illusions?*” As we know, the answer to the visual analog is quick and easy: yes there are visual illusions, and there are many examples at the ready. In my experience, the olfactory question is met with a sense of cautiousness, even confusion, over just what the question itself is asking. Whether there are olfactory hallucinations, however, is met with immediate assurances that there are.

Taking this discrepancy as a datum, I argue that the abstract view of olfactory content can explain the discomfort we have with the notion of an olfactory illusion as well as the apparent comfort we have with its counterpart—the olfactory hallucination. What the abstract view shows us is that, in the case of olfactory experience, the traditional distinction between illusory and hallucinatory experience does not apply. In turn, it directs our attention to a novel notion of non-veridicality—one that has been absent from philosophical discussions of illusion and hallucination.

Traditionally, philosophers have thought that a perceptual experience can misrepresent, or be non-veridical, in one of two ways: the experience can be illusory or it can be hallucinatory. To take a common example, a navy blue sock can look back to you. What you suffer in this case is an illusion with respect to the sock's color. The sock is there, but your visual experience “gets its color wrong”; the experience attributes a property to the sock that the sock does not have. In the case of a hallucination, there is no object there and your experience is not accurate even in that sense. Macbeth famously suffers in just this way; there is no dagger before him and when it appears as though there is, he undergoes a hallucination. Central to the traditional notions of illusion and hallucination, then, is a notion of object-failure; in each, an experience fails in representing a particular object. This much illusion and hallucination have in common. But the nature of that object-failure falls into two kinds. In the case of illusion, a visual experience misattributes a property to an existent object. In the case of hallucination, experience reports that there is an object there, when there is no such object. This difference in the kind of object-failure committed marks what I call the “traditional distinction” between illusion and hallucination.

In order to see why the traditional distinction does not apply to the olfactory case, consider for a moment the visual case. In the case of the typical visual experience, we can ask two separate questions of the object of experience, *o*:

For any property F that o appears to have, does *o* really have F? (*V-Attribution*)

Is *o* there at all? (*V-Existence*)

If the answer to either is “no,” then visual experience fails to present an object accurately. As I put it above, it commits object-failure. But, as we know, they commit object-failure in different ways. If the answer to *V-Attribution* is “no,” my experience misattributes a property to an existent object. And if the answer to *V-Existence* is “no” my experience reports that an object is present when it is not. This difference in the kind of object-failure committed—the difference between visual illusion and visual hallucination—is marked by the different content of *V-Attribution* and *V-Existence*, in what we ask of a given object of experience.

Now consider the olfactory case. If there were olfactory analogs of *V-Attribution* and *V-Existence*, we could ask of an object of olfactory experience, *x*:

For any olfactory property *F* that *x* appears to have, does *x* really have *F*? (*O-Attribution)*

Is *x* there at all? (*O-Existence*)

But, as I have argued previously, olfactory experience only ever reports that there is something or other at a perceiver that is F. This is unlike the visual case where a perceiver's experience typically represents particular objects in one's environment. That is to say, unlike visual experience, olfactory experience is disengaged from any particular object. This is why an object-involving account of its content is unsuitable. In what follows, I will refer to this point as the claim that there are no “presented objects” in olfactory experience^6^.

This explains why we are uncomfortable with the notion of an olfactory illusion. The idea that a smell is misattributed to an object does not grip us and this is because the content of olfactory experience does not support this kind of claim. That is, in olfactory experience, there is no particular thing of which we can ask, as in *V-Attribution*, “it *appears* to be F, but is it really as it appears?” For this reason, I conclude that there are no olfactory illusions^7^.

But, now we are faced with a puzzle. This is because, for the same reasons, there are also no olfactory hallucinations. There is no particular thing of which we can ask, as in *V-Existence*, “yes, it *appears* to be there, but is it?” But, as I have argued, the notion of an olfactory hallucination *is* a notion that we are comfortable with. If what I say about the illusion case is right, however, it ought not to be.

The abstract view of olfactory content can solve the puzzle. As we have seen, the abstract view draws attention to the kinds of questions that we are unable to ask of olfactory experience—namely, questions that refer to particular objects. But, as any account of content will, it also draws attention to the kinds of questions that we are *able* to ask in evaluating an olfactory experience. And, considering *these* questions, I argue, is the key to solving the puzzle.

What questions are we able to ask, then? Given the content of olfactory experience, we can ask of a given olfactory experience and an apparent property F: is there something or other at the perceiver that is (or has) F? In asking this question, we do not pick out any particular object (as olfactory experience does not allow for this). Rather, we ask whether there is *anything at all* around that is F. And, due to its content, a question of this type is the only one we can ask of when evaluating an olfactory experience for veridicality. Notice, however, that this question bears similarities in form to *O-Existence*—the question that is meant to capture a traditional notion of hallucination for olfactory experience. *O-Existence* asks whether a particular object that appears to be F is around; the present question asks whether there is anything around that is F. We do not ask whether F has been misattributed to an object—as we would in *O-Attribution*—but whether F-ness is instantiated at all. The only difference between the present question and *O-Existence* is that it is not a particular object after which we ask. Instead, we ask after a certain property. In each case, however, we ask whether it exists or, better yet, *is there*.

Because of these similarities, I argue that it is understandable that the notion of an olfactory hallucination resonates with us. To be sure, as it turns out it is not the traditional notion of hallucination that does. But it is a notion of hallucination nonetheless—and a novel one at that. As we have seen, when olfactory experience is non-veridical, it incorrectly reports that something or other at the perceiver has a certain property. But this is just to say that when olfactory experience is non-veridical, it incorrectly reports that a certain property is present in the perceiver's environment. As a result, I conclude that the notion of non-veridicality that is suited to olfaction is one of *property hallucination*. It is a notion of misrepresentation, or non-veridicality; but it is one that is disengaged from any particular object. This novel notion of non-veridicality explains two features of the olfactory case. First, it provides the key to understanding why we are comfortable with the notion of an olfactory hallucination, but not comfortable with that of an olfactory illusion. Secondly, in providing a new way of thinking of non-veridicality for the olfactory domain, it also solves the puzzle brought about by the conclusion that there are no olfactory illusions. In particular, it draws attention to reasons for thinking that there are olfactory hallucinations other than those provided by the traditional distinction between illusion and hallucination^8^.

### In support of olfactory illusions: stevenson's view

In what follows, I will take the premises of my argument for granted—in particular, the claim that, in the typical olfactory case, olfactory experience does not achieve figure-ground segregation and, in turn, object-involving status. Recently, Richard Stevenson has responded to my argument that, based on these considerations, there are no olfactory illusions^9^. As we will see, although his embody conclusions of empirical study, Stevenson's own examples of illusion comprise contextual and constancy effects that could, or do, occur in day-to-day olfactory interactions with the world. The empirical studies he cites simply make it clearer that there are such effects. As the point of the present paper is to examine whether Stevenson's cases succeed in overturning my arguments against olfactory illusions in these typical olfactory cases, my and Stevenson's question is the same: are standard cases of non-veridicality for olfactory experience rightly characterized as olfactory illusions?

Stevenson's argument proceeds in two, roughly consecutive stages. First, Stevenson argues that there are olfactory illusions by drawing attention to those cases in which we find them. Secondly, Stevenson examines why the notion of an olfactory illusion has not resonated with us. In this way, his approach is like mine. It is true, according to Stevenson, that we are (or have been) uncomfortable with the notion of an olfactory illusion. Like me, he believes that this is in need of explanation.

Stevenson begins by spending some time discussing the term “illusion” and the kinds of phenomena that it denotes. He tells us that the term “illusion” derives from the Latin “illusio” which, as he cites, has the following meaning: “deceit, to mock or make sport with, the saying of the opposite of what is meant” (1888)^10^. Stevenson takes this definition to involve both an objective and a subjective component. On the objective side, a subject is presented with what is not the case—the “opposite” of what is the case, as the definition states. In this way, the subject is deceived, mocked, or made sport with. But, given that the subject is deceived, she does not notice that there is a disparity between the way the world is and what is being presented to her as the case. Still, she is capable of noticing, Stevenson suggests, given the right kind of circumstances or instruction. This is what Stevenson means by the subjective component of the definition. I take it that it is the term “deception” which “suggests a potential for subjective awareness of [the] disparity” (1888); “illusion,” defined in terms of “deception,” also carries with it that suggestion.

As Stevenson notes, these two aspects of the meaning of “illusion” are not always apparent in the empirical literature on olfaction. Rather, it is the objective component of the term that has currency of use. Although there are subtle differences in the use of “illusion” in the empirical literature, he tells us that, in general, it is used to refer to “a disparity between some objective state of the world and ones [sic] perception of it” (1888). This forms what I will call his *working definition of “illusion.”* This definition, he claims, captures those phenomena that psychologists accept as cases of visual, auditory and somatosensory illusions. Although Stevenson claims that this definition proves enough to pinpoint cases of olfactory illusion, he recognizes that it leaves out any reference to an awareness of the misrepresentation. As he claims, this omission is of little consequence for the cases of visual, auditory and somatosensory illusions. But, as he argues, it has invited the view that there are no olfactory illusions. As evidence of our resistance to the notion of an olfactory illusion, he observes, like me, that the indices of many popular perception textbooks, as well as those of recent specialist books on olfaction, lack any mention of olfactory illusion.

As a way drawing out to the difference between us, then, Stevenson argues that we could take this evidence as indicating one of two things: either (1) that there are no olfactory illusions or (2) that those illusions escape notice. As I outlined above, I argue for (1) and this itself explains our discomfort. As we know, my arguments turn on the traditional distinction between illusion and hallucination together with observations about the phenomenology of olfactory experience. Because olfactory experience is not object-involving, the notion of an olfactory illusion not only has no resonance with us, but also has no application to the olfactory case. Unlike me, Stevenson opts for (2). After arguing that there are cases in which olfactory illusions occur, Stevenson claims that we are typically unaware of having experienced an olfactory illusion, and this accounts for why we might think that there are none. He states this point in terms of verification. We are not only typically unaware that we are undergoing (or have undergone) an olfactory illusion; even if we suspected that we were, we are unable in most cases to verify whether we are (or were) in fact suffering one. Still, as he claims, we would be mistaken to move from this epistemological point to the conclusion that there are no olfactory illusions. Instead, we ought to see our tendency to make this move as the result of a failure to appropriately consider the subjective aspect of the meaning of “illusion” and realize that, unlike their visual, auditory and somatosensory counterparts, olfactory illusions are not the kinds of things of which we are typically aware.

In arguing for (2), however, Stevenson first provides evidence against (1). It is his argument against (1) that I am primarily concerned with in this paper. I will, however, turn to his argument for (2) in my conclusion. At present, I turn to (1).

### Against (1): empirical evidence of olfactory illusions

My discussion of (1) proceeds in two stages, in line with what I take to be the two arguments that Stevenson gives for the existence of olfactory illusions. His first argument forms the bulk of his discussion and involves setting out examples of olfactory misrepresentation that fit his working definition of “illusion.” The second of his arguments occurs in the discussion section of his paper and requires substantial reconstruction. In doing so, we see that Stevenson employs a further notion of illusion—one that, I argue, is the same as the traditional notion that I adopt. Given this, we see that there are two notions of illusion at work in his paper. I will argue that Stevenson is not successful in showing that, in accordance with either of these two notions, there are olfactory illusions.

Let us turn, then, to the first stage of Stevenson's argument. According to Stevenson, what are the cases that we can rightly describe as those of olfactory illusions? Given his working definition of “illusion,” each involves a “disparity” (1888), as he puts it, between the way the world is and one's experience of it. In turn, his arguments assume that there is indeed an objective way that the world is with respect to olfactory phenomena (e.g., quality, intensity, hedonic value), and one that could in principle be accurately represented in olfactory experience. As he puts it: “[a] misperception assumes that there is a veridical state, in which the mind accurately reflects some objective state of the environment” (1893).

According to Stevenson, cases meeting his working definition fall into two categories, each defined by the type of disparity that exists between the external stimulus and a subject's experience^11^. There are the cases in which the same stimulus is experienced differently by a given subject at different times. And there are the cases in which different stimuli are experienced by a subject as the same. According to Stevenson, both of these types of disparity parallel accepted cases of illusion in other modalities^12^.

Let us consider cases of same stimulus-different percept first. According to Stevenson, this category contains a set of cases in which context is thought to affect olfactory experience—in particular, contextual effects of perceived quality, intensity, and hedonic value. In what follows, I will set out several examples of these contextual effects. Stevenson does provide more cases for each category. He also provides examples of variation in the perceived location of a chemical stimulus, as well as an example of an olfactory analog of binocular rivalry. I will set aside these latter two cases. For my purposes, it is enough to consider the perceptual phenomena that fall under the category of “contextual effects^13^.”

In the qualitative category, Stevenson tells us that experiments have shown that the compound dihydromyrcenal is perceived to be more “woody” when smelled in the context of citrus smelling odors, and more “citrusy” when smelled in the context of “woody” smelling odors. In each case, the stimulus remains the same; how a subject perceives that stimulus to be—i.e., the odorant's apparent properties—changes given what other odors it is perceived alongside. If we recall that Stevenson's working definition of an illusion is “a disparity between some objective state of the world and ones [sic] perception of it” (1888), then it would seem that such a case meets this definition. Given that, in each case, the target odorant appears to be “more F,” for some apparent property F, the implication is that there is some way that the target odorant is, irrespective of context^14^. On Stevenson's definition, then, both the “more citrusy” and “more woody” contextual effects constitute illusions with respect to perceived quality.

Stevenson claims that similar effects are reported for perceived intensity and hedonic value. For example, in the case of intensity ratings, experiments have shown that intensity ratings of a range of odor concentrations are affected by intermediate exposure to the same stimulus at weaker, or stronger, concentrations. So, for example, if after having initially rated the intensity levels of a range of odor concentrations subjects are then exposed to a stronger concentration of the same odorant as a biasing task, those subjects later judge the initial concentration range to be less intense. And, as Stevenson tells us, the opposite effect results from intermediate exposure to a weaker concentration. According to Stevenson, this is a case in which there is a disparity between the objective state of the stimulus, as he would put it, and a subject's perception of it. As in the case of perceived quality above, the stimulus remains unchanged throughout the experiment; however, how that stimulus appears to be—that is, its perceived intensity—changes given the context of perception, in this case one created by the biasing task. The suggestion is that, prior to the biasing task, there is no disparity between the intensity properties of the stimulus and the subject's perception of them. It is only after the biasing task that the subject suffers an illusion with respect to the intensity of that stimulus.

Finally, in the category of hedonic judgment, Stevenson cites a series of experiments in which labels reflecting positive and negative contexts have been shown to affect judgment of the pleasantness of an odorant stimulus. As he tells us, in a particular experiment, previous exposure with the label “toilet cleaner” (i.e., a negative context) affects the judgment of a pine odor's pleasantness in later contexts labeled “Christmas tree” (i.e., a positive context). Similarly, initial exposure to the same odorant with the label “Christmas tree” affects judgment of its pleasantness in later context labeled “toilet cleaner.” In the first case, perceivers judged the shift in pleasantness to be less than they did in the second case, when the labels were reversed. This is despite the odorant stimulus remaining constant throughout. Verbal labels, then, can affect judgments of pleasantness.

Although Stevenson does not state this explicitly, these are, for him, cases of illusion because of the relation that experience bears to our hedonic judgments. In particular, the case suggests that those judgments are made on the basis of experience such that a difference in judgment indicates a difference in the associated olfactory experience. It is only if this is true that differences in hedonic judgment could tell us anything about the existence of illusions in the olfactory case. For illusions are cases of perceptual misrepresentation, as Stevenson claims earlier; they cannot only be matters of inaccuracy of judgment—although, if we take our illusory experiences at face value, our judgments will be inaccurate as well. With this in mind, it is clear that, for Stevenson, cases of variation in hedonic judgment involve a disparity between some objective state of the stimulus and a subject's perception of it. The stimulus remains the same, after all. To be sure, in the experiment he cites, this disparity might underlie each of the subject's initial judgments, given that in both cases the odorant is perceived with verbal labels. It might be that “the veridical state, in which the mind accurately reflects some objective state of the environment” (1893) is one had in the absence of any verbal label. (And, prima facie, this seems plausible). Despite this, even double disparity in this case shows that, on Stevenson's working definition, there are cases of olfactory illusion. That is, if both labeling cases are ones of disparity, then so much the better for his argument that there are olfactory illusions^15^.

Now to cases of different stimulus-same percept. In this category, Stevenson cites two instances of perceptual stability, or constancy phenomena. The first example involves intensity. According to Stevenson, research has found that variations in the flow and, in turn, concentration of an odorant over the olfactory epithelium is registered by neural responses of the olfactory nerve. Despite this, such variation does not arise at the level of experience. Rather, despite variation in the concentration of an odorant passing over the olfactory epithelium, subjects perceive odor stimuli as relatively stable with respect to intensity. Stevenson suggests that these results show that the epithelium is not only sensitive to the stimulus itself, but to the rate of airflow over it. Due to this added sensitivity, the olfactory system adjusts for variations in concentration relative to changes in airflow. The result is constancy with respect to the perceived intensity of the stimulus. Given Stevenson's working definition of “illusion,” we have a case where there is disparity between the objective state of the stimulus and the nature of the experience resulting from it. In this case, we have a difference in odorant concentration that fails to show up at the level of experience. This subdued sensitivity to differences in an odorant stimulus amounts to an illusion, Stevenson suggests, because a veridical experience of it would represent its actual concentration (presumably in the form of what we call intensity of olfactory quality). Because that actual concentration is not represented at the level experience, Stevenson indicates that atleast some of our representation of concentration is illusory^16^.

Stevenson's second example involves constancy in perceived quality despite differences in, or changes to, the chemical constitution of an odorant stimulus. Drawing on work he presents in Wilson and Stevenson (2006, 2007), Stevenson tells us that degraded input, or varying formulations of a stimulus at the receptor site, can be completed at the level of experience. Because of the complexity of the olfactory environment, one might not receive information about all of the components of a certain odor stimulus, for example coffee, and yet still be able to smell that that coffee is present. What accounts for this ability are prior encodings of odorant stimuli in the form of stored templates of patterns of receptor excitation in the olfactory cortex. As Stevenson claims, a “perfect fit” (1892) between input and template is not required; rather the olfactory system is able to recognize certain sub-patterns of receptor activation against existing templates of activation. The result is, however, not a “partial” experience of coffee; it is an experience of coffee. Without these templates, Wilson and Stevenson (2006, 2007) claim, it is unclear how such constancy might be achieved. Like constancy of intensity, then, it would seem we have a case where there is disparity between the objective state of the stimulus and the nature of the experience resulting from it. In this case, we have a difference in chemical constitution that fails to show up at the level of experience.

In sum, Stevenson alleges that all of the cases of same stimulus-different experience and different stimulus-same experience involve misrepresentation and, in particular, illusion. He argues that each case involves a circumstance in which there is a disparity between some objective state of the world and a subject's experience of that state. In accordance with his working definition of “illusion,” then, these are all cases of illusion.

### Olfactory illusions?

In what follows, I will take for granted that each of these cases is one that we can assess for veridicality. I will also take for granted that there is some objective state of the world that our olfactory experience is capable of misrepresenting and does so in each of these cases. Given these assumptions, I want to now consider whether, or how, Stevenson's arguments affect my own.

As a way of making headway on these questions, it is important to first note that my notion of non-veridicality could handle these cases of alleged illusion^17^. Recall that my notion of non-veridicality involves the consideration of whether, for a certain olfactory feature F, there is anything at all at the perceiver that is F. So, to take the case of dihydromyrcenal as an example, evaluating the “more woody” case for veridicality involves asking whether there is anything at all at the perceiver that has, objectively, that degree of woodiness. Or, as I have also put it, it involves simply asking whether, in those perceptual circumstances, that degree of woodiness is instantiated. If the answer is “no,” then the experience is non-veridical. As I am assuming with Stevenson, that degree of woodiness is not instantiated at the perceiver—there is nothing at all that is “more woody” at the perceiver. In this case, then, the answer to my question is “no,” and one's experience in this circumstance counts as non-veridical.

Notice, however, that my notion of non-veridicality for olfactory experience is no different than Stevenson's notion of illusion. Remember that, according to Stevenson, an illusory experience involves “a disparity between some objective state of the world and ones [sic] perception of it” (1888). But this is just what, on my notion of non-veridicality for olfactory experience, a non-veridical experience involves. To consider whether F-ness is instantiated at a perceiver is to consider whether the perceiver's experience “accurately reflects some state of [her] environment” (1893). If it does not, then there is a disparity between that state of the environment and a perceiver's experience of it. To return to the case of one's experience of the woodiness of dihydromyrcenal, Stevenson's notion of illusion requires that we ask whether that degree of woodiness is instantiated by some state of the environment, where “environment” presumably denotes the space around the perceiver eligible for inhalation^18^. But my notion of non-veridicality asks the same—that s, whether that degree of woodiness is instantiated at the perceiver. Given what Stevenson has told us, then, “Does S's experience of F-ness accurately reflect some state of the environment?” amounts to asking “Given that S has an experience of F-ness, is F-ness instantiated at the perceiver?” Just like Stevenson's notion of illusion, my notion of non-veridicality does not ask after any particular thing that appears to be F. Rather, in asking whether anything at all instantiates F-ness, it asks whether, to use Stevenson's terms, there is a state of the environment in which F-ness is instantiated.

As it stands, then, Stevenson's working notion of illusion fails to address my arguments against olfactory illusions. Both of us provide the same analysis of his cases. But if we truly disagree, then we ought to provide different analyses of them. At this point, then, any purported disagreement between us amounts to a mere difference in terminology. He calls his cases of disparity illusions, while I do not. But, other than that label, our characterizations of them amount to the same. Because of this, if Stevenson is to refute my arguments, he must do more to address them directly.

I hinted at what else is required above when I claimed that, because my notion of non-veridicality does not ask after any *particular thing* that appears to be F, it amounts to the question of whether there is a state of the environment in which F-ness is instantiated. My conclusion that there are no olfactory illusions hinges on the observation that olfactory experience is not object-involving, that there are no presented objects in olfactory experience. Recall that, on that traditional way of categorizing non-veridical experience, both illusion and hallucination involve what I call object-failure—that is, a failure to represent a particular object accurately. If there are no presented objects, then that categorization fails. And, as I argue, there are no such objects. This is because the very nature of olfactory experience—its “smudginess,” as I have put it—doesn't allow for a distinction between figure and ground. These considerations of phenomenology constitute my reasons for denying that there are olfactory illusions. What is required for Stevenson to address my arguments, then, is an argument for the conclusion that, in the cases of alleged illusion he cites, there *is* a presented object that appears to be other than it is.

Stevenson appears to argue for just this in his later discussion section—although he does not turn back directly to his example cases. Before moving on to these arguments, it is important to note some potentially misleading claims that Stevenson makes when introducing this discussion. After presenting his alleged cases of olfactory illusion, Stevenson claims that “the apparent actuality of olfactory illusions would seem to call into question Batty's (2010b) claim that olfactory experience lacks object status” (1895). As it stands, this claim is far too quick. It carries with it the implication that Stevenson has discussed his cases of olfactory illusion in terms of presented objects. But he does not make any claim of the sort, focusing instead on states of the environment. But, as we have seen, casting these alleged cases of illusion in terms of mere states of the environment is not enough to address my arguments. As it stands, then, “the apparent actuality of olfactory illusions” does not “call into question Batty's (2010b) claim that olfactory experience lacks object status” (1895)^19^. As I claimed above, more needs to be said to establish this claim.

Stevenson then seems to recognize this when he goes on to claim that olfactory experiences do in fact achieve “object status” (1895). Although he cites other authors who have claimed that olfactory experience achieves object status, it is most helpful to consider what Stevenson himself has argued with respect to this claim. Wilson and Stevenson (2006, 2007) argue for an object-based model of theorizing about olfaction, a model they call the Object Recognition Model (from hereon ORM). In particular, they argue that olfactory experiences represent “olfactory objects.” Given that they also refer to these objects as “odor objects,” it is safe to assume that, on the ORM, the objects represented in olfactory experience correspond to odors—or, collections of volatile molecules in a perceiver's environment. One of their common examples is the “coffee object.”

Returning to a type of view about content that I discussed in section one, we will see that the ORM suggests that olfactory experience is object-involving—that is, that it represents that a particular object is present in your environment as opposed to some object or other, as my abstract view maintains. In turn, this suggests that Stevenson's notion of illusion at this point of his paper is in fact the more robust, traditional notion rather than the “working definition” that he relies on previously. If olfactory experience is object-involving, then it is eligible for misrepresentation in both of the traditional ways. In particular, to return to a previous question, we *can* ask of an object of olfactory experience, *o*:

For any property *F* that *o* appears to have, does *o* really have *F*? (*O-Attribution)*

That is, there is some particular thing of which we can ask, as in *O-Attribution*, “it appears to be F, but is it really as it appears?” But *O-Attribution* is the question that captures the traditional notion of illusion. If the ORM is true, then, my claim there are no olfactory illusions is shown false.

What are we to make of the ORM? If the ORM is to encompass a successful response to my argument against olfactory illusions, then olfactory experience must single out objects in the requisite way—that is, it must be object-involving. As a way of understanding why Wilson and Stevenson think it does, it is important to look briefly at the traditional model of theorizing about olfaction that their ORM aims to replace—and why it does so. They call this model the Stimulus Response Model (from hereon, SRM). Given the history of scientific theorizing about olfaction, we can extract two core claims of the SRM. First, the SRM assumes that olfactory experience is *analytic*—that is, those features of a chemical stimulus that trigger receptor excitation will map onto features of the resulting experience. In other words, the SRM claims that, in some important sense, olfactory experience can be “broken down” into those initial features of the stimulus and/or receptor types sensitive to those features. Secondly, and relatedly, the SRM assumes that a characterization of olfactory experiences is exhausted by an account of how the particular features of the stimulus and/or receptor site are presented in experience. On the SRM, no appeal to objects is necessary to provide that characterization.

According to Wilson and Stevenson, the SRM proves unsatisfactory because olfactory experience doesn't live up to the standards that the SRM sets for it. This is because olfactory experience is, as they tell us, largely synthetic. That is to say, rather than producing an experience of an array of discriminable properties, the various properties of the stimulus produce a largely irreducible experience—a “wholistic unitary percept” (2007, 1821), as they put it. One particularly telling way that they deliver this point is by asking us to consider the complexity of the average odorant stimulus. Much of what we encounter with our noses are chemical mixtures. The coffee odor, for instance, consists of over 600 volatile compounds that together give rise to what we might call the “coffee experience.” It is a distinctive experience—one that gets us up in the morning. But it is not an experience in which we are able to discriminate anything close to the number of causally efficacious components of the stimulus responsible for it. As it's been noted in the empirical literature, it is now commonly accepted that even the experts are only ever able to distinguish two or three of the major components that constitute a given odor. So, while the coffee stimulus has a remarkable complexity, it does not have a *perceived* complexity^20^. Compared to the complexity of the stimulus itself, the coffee experience is simple. It's just *of coffee*. But this is not the way that our experience of the coffee odor should be if the SRM is true. Although, as Wilson and Stevenson concede, olfactory experience can fail to be wholly synthetic, if it were analytic, our experience of the coffee odor would be different than it in fact is. We might think that, if the SRM were true, there would be no such thing as *the* coffee smell *per se*—just an array of apparent properties. But there is. Given this, the SRM fails to capture the phenomenological facts of our experience. Wilson and Stevenson therefore conclude that it is a misguided model and must be rejected.

In place of the SRM, Wilson and Stevenson propose the ORM. We already know that such a view is object-based, that olfactory experience represents “olfactory objects,” or “odor objects.” We also know that it is safe to assume, given to their name, that these objects correspond to odors in our environment. But, what are these perceptual objects? Or, to put it another way, in what sense do odors in the environment show up at the level of experience? Their criticism of the SRM provides the answer to this question. According to Wilson and Stevenson, odors show up as those “wholistic unitary percepts” (2007, 1821), as the synthetic percepts that the SRM fails to predict. The “coffee object,” then, is that largely synthetic percept that results from sniffing the coffee odor.

Now, it is not simply because olfactory experience is largely synthetic that Wilson and Stevenson claim it is object-involving. It is rather what it can achieve as a result of its being synthetic that they claim secures the view. According to Wilson and Stevenson, the “defining feature for [perceptual] objecthood” (2007, 1823) is figure-ground segregation, and they argue that olfactory experience can achieve just that^21^. Their reasons for thinking so draw on similar considerations as those of Stevenson's case of constancy of perceived quality^22^. In order to draw attention to how olfactory experience achieves figure-ground segregation, Wilson and Stevenson ask us to consider the complexity of our olfactory environment. At any given moment, we are barraged with volatile molecules given off by the various things in our environment. Insofar as almost everything in our environment gives off these molecules, we can say that *everything smells*. And a remarkable number of those molecules make their way to the olfactory epithelia with every intake of breath. Despite this, our olfactory system is able to achieve the most impressive of discriminatory feats. In the midst of the “confusion” of our olfactory environment, as they put it, we are able to smell *coffee*. The “wholistic unitary percept” (2007, 1821) *coffee* is an apparent figure, one that stands out in the midst of a complex, and noisy, background. This “experiential prominence” in the midst of that noisy background is what Wilson and Stevenson refer to as figure-ground segregation.

It must be noted, however, that, unlike the visual case, Wilson and Stevenson claim that figure-ground segregation is achieved aspatially. According to Wilson and Stevenson, olfactory experience is, in and of itself, aspatial. To return to our previous example, the coffee object is an apparent object—just not one that is presented in space. Still, according to Wilson and Stevenson, given experiential prominence and, in turn, the achievement of figure-ground segregation, it is an apparent object nonetheless. After all, figure-ground segregation is, for them, the defining feature of perceptual objecthood and, if correctly characterized as such and achieved, constitutes the presentation of an object. Wilson and Stevenson agree with me, then, in an important respect—namely, that spatial figure-ground segregation is not something that applies to olfactory experience. Other than myself and Stevenson's common focus on standard olfactory experiences, then, there is an additional point of agreement between us. But is this enough to show that, in such cases, olfactory experience presents objects and, in turn, is eligible to be illusory?

As a way of answering this question and in order to compare our respective views, we need to say something more about the ORM. According to Wilson and Stevenson, underlying experiential prominence is the template mechanism that I referred to earlier, in my discussion of Stevenson's case of constancy of perceived quality^23^. Wilson and Stevenson argue that, over time, the olfactory system builds up a store of templates in the olfactory cortex of patterns of receptor input. Once stored, these templates allow the system to recognize those patterns against variable arrays of receptor input. In turn, this kind of processing endows us with important discriminatory abilities such as the ability to smell coffee although there are other smelly things about. Contributing to these achievements, then, are learning and memory. In short, the growing store of templates constitutes learning; drawing on those templates in processing olfactory information amounts to the execution of memory^24^.

If experiential prominence is rightly characterized as figure-ground segregation, then Wilson and Stevenson's view is one according to which olfactory experience is object-involving. This is because the very nature of figure-ground segregation is such that it allows a perceiver to single out a particular object in her environment. We must now consider whether experiential prominence demonstrates that olfactory experience is object-involving and, in turn, secures the claim that it achieves figure-ground segregation.

It is not clear that experiential prominence establishes this. The problem lies in the fact that my view is consistent with all of the phenomenological data that Wilson and Stevenson cite. In order to see that this is so, let's return to the coffee example and look at what my view of olfactory representation is able to say about this case. On my view, when we smell the coffee, there is a distinctive property, or set of properties, presented to us in olfactory experience. I will also grant that, in certain circumstances, that property, or set of properties, stands out from other properties instantiated in a perceiver's environment—namely in those circumstances in which we smell coffee. Given the complexity of the olfactory environment, and the way that olfactory experience is given those facts, it would be foolish to deny this experiential prominence. Moreover, I can also grant Wilson and Stevenson's claim that, in olfactory experience, such prominence is achieved in virtue of a relative match between stored templates in the olfactory cortex and patterns of receptor excitation. Where my view will differ from Wilson and Stevenson's is in what the result of that template matching is—that is, in what that experiential prominence amounts to. On my view, it amounts to the presentation of a property, or a small set of properties presented together as a result of that template matching^25^. This much is in keeping with Wilson and Stevenson. But, unlike what Wilson and Stevenson claim, that those properties “show up” at the level of experience indicates the presence of some object—just not any object in particular.

Notice that, at this point, I have granted all of the perceptual data that Wilson and Stevenson cite in favor of figure-ground segregation. In doing so, I stop short of positing that the presentation of those properties, as distinct in a complex environment, amounts to the presentation of a particular object. But, again, it does not stop short at the expense of any of the perceptual data that Wilson and Stevenson cite in favor their view. In particular, and most importantly, that data that they take to be indicative of figure-ground segregation is accounted for without taking that step.

What this shows is that it isn't clear that experiential prominence is best characterized as figure-ground segregation. This is because, as a comparison with my view has demonstrated, Wilson and Stevenson haven't shown that it is an apparent *figure* that shows up at the level of experience. But demonstrating that there is such a figure—or object—is exactly what is required in order to establish that the more robust notion of illusion is one that can occur in olfactory experience. To return to our previous question, Stevenson must establish that *O-Attribution* is a question that we can ask of olfactory experience. But his own “object-based” view of olfactory experience does not. Given this, he fails to demonstrate that my claim there are no olfactory illusions is false.

It is important to note that responding to present worries about ruling out my abstract view requires more that simply drawing attention to the fact that there exist patterns of excitation at the receptoral level, nor to the fact that that such patterns are stored in long-term memory to expedite later olfactory discrimination. What is at issue is whether these patterns and combinations show up, at the level of experience, as perceptual objects. The question is whether the experiential output of template matching—Wilson and Stevenson's “wholistic unitary percepts” or “synthetic odor objects”—ought to be characterized in object-involving terms. And it isn't clear that there are the materials with which to adjudicate between that kind of view and mine—at least if we are relying on observations of experiential prominence to decide it.

Are we now left at an impasse, with each of us able to account for the relevant data and nothing left to adjudicate the issue? I think that we are not. I grant that figure-ground segregation allows us to single out a particular object in our environment. That is, I grant that figure-ground segregation forms the basis of object-involving content. Wilson and Stevenson agree. But they also assume something stronger than I do: that if the distinction is to apply in the realm of olfaction, it must apply non-spatially. But not only has this revision of the concept proven problematic, it also deprives us of the ordinary spatial notion of figure ground, a notion which we *do* need—just not for humans. To see that this is so, compare our olfactory experiences to those of other animals. The hammerhead shark, for example, enjoys a sense of smell that is directional. Given its extremely wide head, a stimulus coming from the extreme left of the hammerhead's head will arrive at the left nasal cavity before it does the right. If the stimulus is blood, the hammerhead's response is instantaneous— it turns in the direction of its source. I take it that we are quite happy to admit that the hammerhead represents the location of a food source, much in the same way that we are able to represent, via audition, the location of a “bang” outside. In the latter case, we are happy to admit that auditory experience achieves figure-ground segregation—and does so spatially. Given this, it is plausible to conclude that the hammerhead also achieves the same in its olfactory experience. That is to say, the hammerhead shark is a creature that enjoys spatial figure ground representation and thus object-involving olfactory content. Clearly we are not like the hammerhead, as Wilson and Stevenson admit. But it would be strange to conclude that the hammerhead's olfactory experiences are to be evaluated according to one notion of figure-ground segregation, while ours are not. If we are to account for the difference between us and the hammerhead, then, we require the spatial notion of figure-ground segregation.

What this shows is that the spatial notion of figure-ground segregation remains useful in the olfactory case. We can make distinctions with it that we need to make—for example, we can explain the difference between us and the hammerheads. What's more, it allows for a unified notion of figure-ground segregation across the sense modalities. In those types of experience in which we think of figure-ground segregation as achieved—vision, audition and touch, for example—we do so on the basis of the richness of its spatial representation. In those types of experiences in which we worry whether, or wonder if, figure-ground segregation is achieved—arguably olfaction and taste—I take it that we so on the basis of the observation that those types of experiences are not as spatially rich as those where we grant happily that there is figure-ground segregation. What this suggests is that figure-ground segregation forms a kind, one defined by the type of spatial representation achieved by an experience.

If, as I have argued above, we ought to evaluate olfactory experience in accordance with this notion of figure-ground segregation, then we ought to accept my abstract view. And, if we accept that view, then we are committed to accepting three further things. First, we are committed to accepting my analysis of experiential prominence over Wilson and Stevenson's, driven as mine is by the abstract view of olfactory content. Second, and relatedly, we ought to accept my conclusion that there are no olfactory illusions. Finally, given the accuracy conditions set forth by the content of olfactory experience, we ought to accept that the appropriate notion of non-veridicality for the olfactory case is one of property hallucination.

Now Stevenson says little about the notion of property hallucination *per se*, focusing instead on the negative stage of my 2010b argument that there are no olfactory illusions. Still, let me say something further about the benefits of adopting a notion of property hallucination and of a non-object based notion of non-veridicality. Scientists and philosophers alike have long been interested in non-veridicality, or perceptual misrepresentation. But it has also been assumed that non-veridicality falls into one of two categories—illusion and hallucination. As I noted in section 1, these ordinary notions each involve the misrepresentation of objects, or “object-failure,” as I have called it. It is true that, with property hallucination, I am also talking about non-veridicality. But what is interesting about property hallucination is that it is a form of non-veridicality that current accounts of non-veridicality do not allow for, focused as they are on the representation of particular objects. Drawing attention to property hallucination, then, identifies a new category of non-veridicality. Given that both scientists and philosophers have been interested in the information putatively conveyed in olfactory experience, and the nature of the ways in which experience may misinform a subject, the introduction of property hallucination presents a novel way of thinking about, and categorizing, olfactory misrepresentation.

But the interest of property hallucination for olfaction is not only restricted to the olfactory case. It is also helpful in driving further thinking about perceptual experience in general. That is, its introduction forces us to re-think the nature of veridicality and non-veridicality more generally across all of perceptual experience. For example, the notion of property hallucination opens up the possibility that there are cases in other modalities that are best characterized as those in which we do not perceive particular objects but only certain properties, and that this novel notion of non-veridicality best accounts for those cases. One case that I have discussed previously is the visual experience of looking at a uniformly colored expanse^26^. To be sure, this is not a typical visual experience, as I argue the analog case for olfaction is; but it is one that, if in fact a misrepresentation of color, is plausibly categorized as a case of property hallucination. A third category of non-veridicality, then, is incredibly interesting because it allows us to look deeper at the experiences of other modalities, comparing and contrasting the ways in which experiences in those can mislead.

Finally, adopting my third category of non-veridicality directs our attention to the possibility that there might be even further categories of non-veridicality—whether these other, previously unconsidered notions turn out to be categories in their own right, or sub-species of those we already adopt. Not only, then, does my notion of property hallucination introduce a new category that we previously lacked in describing perceptual misrepresentation; it also directs attention to the possibility that our account of non-veridicality might be lacking in further, equally interesting, ways. And this further result, I take it, would be interesting for philosophers and scientists alike.

## Conclusion

Earlier I promised to say something further about what I labeled Stevenson's (2), namely his claim that olfactory illusions typically escape notice. Obviously I disagree that they do. I argue that there are no olfactory illusions and so there is nothing in this case to escape our notice. Still, my abstract view of content can explain why we might think, like Stevenson claims, that the difference between olfaction and other modalities, “relates to issues of verification (i.e., ones [sic] capacity to independently confirm what one is smelling” (1888). To take the case of vision as an example, it is easy to see how we are able to verify what we seem to see. In the case of visual experience, because we are able to discriminate individual objects, we are able to ask, and in principle capable of verifying, whether *that object* is in fact in the scene before our eyes. Given that it is presented as such, we are also in principle capable of verifying whether the properties it appears to have are those that the object in fact has. In each case, we go out and explore the environment; we go to that object that we appear to see and “interrogate” further. These two capacities for verification are implied by our previous two questions about misperception, *V-Existence* and *V-Attribution*.

But, as I have argued, the olfactory analogs of each—*O-Existence* and *O-Attribution*—do not in fact apply to olfactory experience. This is because there are no presented objects in olfactory experience; olfactory experience is not object-involving. It is unclear, then, how we are able to verify what we smell. Like the visual case, we may very well explore our environment further; but it is not the case that we are able to pinpoint that object we appear to smell and “interrogate” it further. The most we are able to do is locate those properties we appear to smell, to determine if it is in fact what we thought it was, or if it appears to be elsewhere around us. But notice that this is just to ask after whether a property, or set of properties, is instantiated in the environment. It is not to ask after any particular object.

It is no wonder, then, we feel suspicious about our abilities to verify our olfactory experiences. We simply are unable to do so in the same way as we are in the visual case. But, unlike what Stevenson claims, this difference is a result of the fact that there are no presented objects. In fact, if we take Wilson and Stevenson at their word, then it would seem that we *would* be able to verify what we smell in the much stronger sense of “verification” present in the visual case. That is, we ought to be able to pinpoint a particular object in our environment and ask after it. But we cannot. Not only, then, is abstract view vindicated with respect to its claims about olfactory illusions; it is also able to explain those considerations about verification that, as it turns out, Stevenson himself is unable to accommodate.

### Conflict of interest statement

The author declares that the research was conducted in the absence of any commercial or financial relationships that could be construed as a potential conflict of interest.

## Footnotes

^1^It must be noted that all of my previous arguments concern human olfaction. I will have something to say about the olfaction of other creatures at the end of the paper.

^2^For example, both Peacocke (1983) and Lycan (1996, 2000) suggest that the phenomenology of olfactory experience does not uphold a representational view. In the opening chapter of his Sense and Content (1983), Peacocke suggests that “a sensation of [smell] may have no representational content of any sort, though of course the sensation will be of a distinctive kind” (3). This is all he has to say, however. Still, his remarks suggest a sensational view of olfactory experience. Echoing Peacocke, Lycan claims that “phenomenologically speaking, a smell is just a modification of our consciousness, a qualitative condition or event in us” (2000, 281), “lingering uselessly in the mind without representing anything” (1996, 245). Lycan does go on to argue that olfactory experience is representational; but it is clear from these remarks that he thinks that we cannot uphold such a view on the basis of the phenomenology of olfactory experience. He, in turn, proposes that the appropriate notion of content for olfactory experience is a teleological one (1996).

^3^These examples all show that olfaction cannot solve the Many Properties Problem—that is, the problem of distinguishing between scenes in which the same properties are instantiated, but in different arrangements. Vision can solve this problem, and it does so by grouping perceptual features together in space. This grouping amounts to the presentation of sensory individuals. Olfaction, I argue, does not achieve this kind of perceptual grouping. For these examples, see Batty (2010a, 2011).

^4^As I cite in my previous paper (Batty, 2010b), studies suggest that that humans are able to achieve some measure of spatial discrimination in highly controlled laboratory settings. See, for example, Porter et al. (2005) and von Békésy (1964).

^5^Unless it is important to otherwise note, in what follows, my use of “olfactory experience” or “olfactory experiences” will denote those typical, day-to-day, olfactory experiences.

^6^Although we might say that, on the abstract view, olfactory experience presents objects, I intend “presented objects” and its counterpart “presents objects” to denote circumstances in which olfactory experience presents particular objects, as an object-involving view of its content would have it.

^7^Note that it will not help here to argue that sometimes physical objects (“source objects,” as we might call them) seem to have properties that they do not in fact have. My claim is that, given the nature of the phenomenology of olfactory experience, we are never in a position to know what particular object has, or is the source, of the properties that we perceive. That is to say, while olfactory experience predicates properties of “something or other,” it is otherwise silent on the nature of that object—whether it be, in fact, an odorous effluvium or a “source object.” Interrogating olfactory experience further will not tell us what olfactory objects are. So, although we do attribute—and at times incorrectly—properties to source objects, we do not do this on the basis of olfactory experience alone. Arguably, when we do, we do so on the basis of a network of background beliefs about source objects gained from past experience and/or the exercise of other modalities in discovering those sources. Again, those source objects are not revealed to us in olfactory experience itself and, as a result, any mistaken attribution to them we make does not provide a counterexample to my conclusion.

^8^One might worry that my claim that non-veridical olfactory experiences are best characterized as property hallucinations blurs certain intuitive distinctions that we make. For example, consider the two following cases: (1) a case in which there is no odorant at all in the room, and yet you smell coffee, and (2) a case in which there are only dry flowers in the room but in which you misrepresent their smell as coffee. On my view, the experiences of each would both count as property hallucinations. They are each cases in which, on the abstract view, the content of their respective experiences will be the same. And, in turn, in evaluating the veridicality of each, all we can ask is “is the coffee smell instantiated?” Still, just because the content of olfactory experience does not distinguish between a case in which we have an odorant, or odorant source, and one in which we do not, this is not to say that we cannot maintain the intuitive difference between these two cases. It remains open to explain that difference as a result of inference from past experience, background beliefs as well as the contribution of other sense modalities—the latter, in particular, for the case of (2). See also fn. 7 for a related point.

^9^Stevenson does not directly address my notion of property hallucination. Given that my arguments for property hallucination in the olfactory case turn on my arguments against the existence of olfactory illusions, we can interpret his failure to do so as resulting from his denial of my conclusion regarding olfactory illusion. If there are olfactory illusions as tradition would have them, then there is no need to posit a novel notion of non-veridicality for the olfactory case. I will, however, return to the benefits of this novel notion later in the paper.

^10^All references to Stevenson will be to Stevenson (2011).

^11^In discussing Stevenson's examples, I adopt his use of “disparity” to refer to that difference between the way things appear and the way that they are. It is a term that is rarely used in the philosophical literature, with philosophers often adopting characterizations in terms of the inaccuracy of a representation.

^12^I will avoid going into the details of these illusions in other modalities. For present purposes, it enough to note that he thinks that there is this parallel.

^13^I set aside cases of perceived location and binaral rivalry for reasons other than brevity. To give Stevenson's discussion of olfactory localization full treatment would involve dealing with difficult questions regarding the status of the retronasal as truly olfactory. Given that my claims regarding olfactory illusion center on orthonasal olfaction, I consider only the orthonasal. I set aside his consideration of binaral rivalry because it isn't clear that it constitutes an illusion, even in his working sense. In the case of binaral presentation, one's olfactory experience switches back and forth from the presentation of an odor located discretely at one nostril to an odor located discretely at the other. In each case, the odorant is indeed at the nostril at which one's experience represents it as being. What one's experience does not represent is that there is another odorant present at the other nostril. (Assume that experience gets the quality and intensity “right.” He does not claim that there is any other disparity that that of localization.) But surely in each case (switching from one nostril to the other) one's experience “accurately reflects some objective state of the world” (1888)—namely, that a certain odorant is located at a certain nostril. What it does not report is that there is an additional odorant located at the other. But this is just a failure to perceive something in one's environment. By Stevenson's own lights, the experience hasn't conveyed any information that is false; it has simply failed to convey all of the information about the perceiver's environment. Accurately representing some objective state of the environment does not involve representing every feature of that environment. That is too strict a constraint on veridicality—arguably one that we would never meet. What matters for determining whether an experience is veridical is whether what experience does represent is represented correctly—i.e., veridically.

^14^In line with Stevenson's characterization of illusion, I take it that this is a feature of the odorant that could in principle be represented veridically in olfactory experience. In what follows, I will leave out reference to these counterfactual circumstances. But it should remain understood that, according to Stevenson, they could obtain.

^15^Stevenson cites similar experiments in which a target stimulus is judged to be more pleasant if presented with odorants that are typically judged to be less pleasant, and less pleasant if presented with odorants that are typically judged to be more pleasant. Again, it must be that, for Stevenson, underlying cases of variation in hedonic judgment is a disparity between some objective state of the stimulus and a subject's experience of it. If this is true, these cases also constitute illusions on his working definition of “illusion.”

^16^Given that Stevenson presents these as relatively common instances of perceptual constancy, it might turn out that much of our representation of concentration is illusory. It is unclear whether this is something that Stevenson would be happy to accept. One way to avoid that result would be to claim that olfactory experience represents concentration relative to air flow over the epithelium. In this case, our judgments of intensity would be more eligible for accuracy at the level of experience. I leave this proposal, however, for another time. The important point is that it is not a proposal that Stevenson wishes to entertain, opting instead for claims of illusion in these cases.

^17^In what follows, I will simply refer to my notion of non-veridicality for the olfactory case, as opposed to my notion of property hallucination for it. Given that I argue that the latter is the only way that (human) olfactory experience can be non-veridical, there is no room for confusion here.

^18^If “environment” denoted anything greater, then we would have to count as veridical cases in which there is nothing at a perceiver that is F, although there appears to be, but in which there is something “farther out” in the perceiver's surroundings that is F—although the perceiver does not take any of that odor in. Presumably we want to still count these cases as non-veridical.

^19^Strictly speaking, I do not deny that olfactory experiences lack object status. I argue that olfactory experiences represent objects, just not particular objects, and not in a way that allows for olfactory illusion. That is, I argue that olfactory experience is not object-involving. Given this, I will assume that by “lacks object status” Stevenson means “is not object-involving.”

^20^This would explain why we are surprised to hear of the complexity of the coffee odor.

^21^According to Wilson and Stevenson (2007), they adopt this definition from Kubovy and Van Valkenburg (2001).

^22^See page 6 of this paper.

^23^Again, see page 6 of this paper.

^24^Wilson and Stevenson say much more about the physical mechanisms underlying what I have referred to as “template mechanisms.” For my purposes, it is enough to provide a model of their view.

^25^Here I am not claiming that olfactory experience achieves the perceptual grouping required to solve the Many Properties Problem. I am simply, for the sake of comparison, adopting Wilson and Stevenson's claim that, in some cases, we are able to distinguish two or three components of an odorant stimulus. While they claim that, even in these cases, we are presented with olfactory objects, I here claim that a view that denies that there are such objects can accommodate the data they cite. It is important to note that amongst the data they cite is not the claim that olfactory experience can report on different arrangements of those properties along some dimension—e.g., the spatial dimension. But it is this kind of achievement that underlies the ability of a sensory system to solve the Many Properties Problem.

^26^See for example, Batty (2011).
